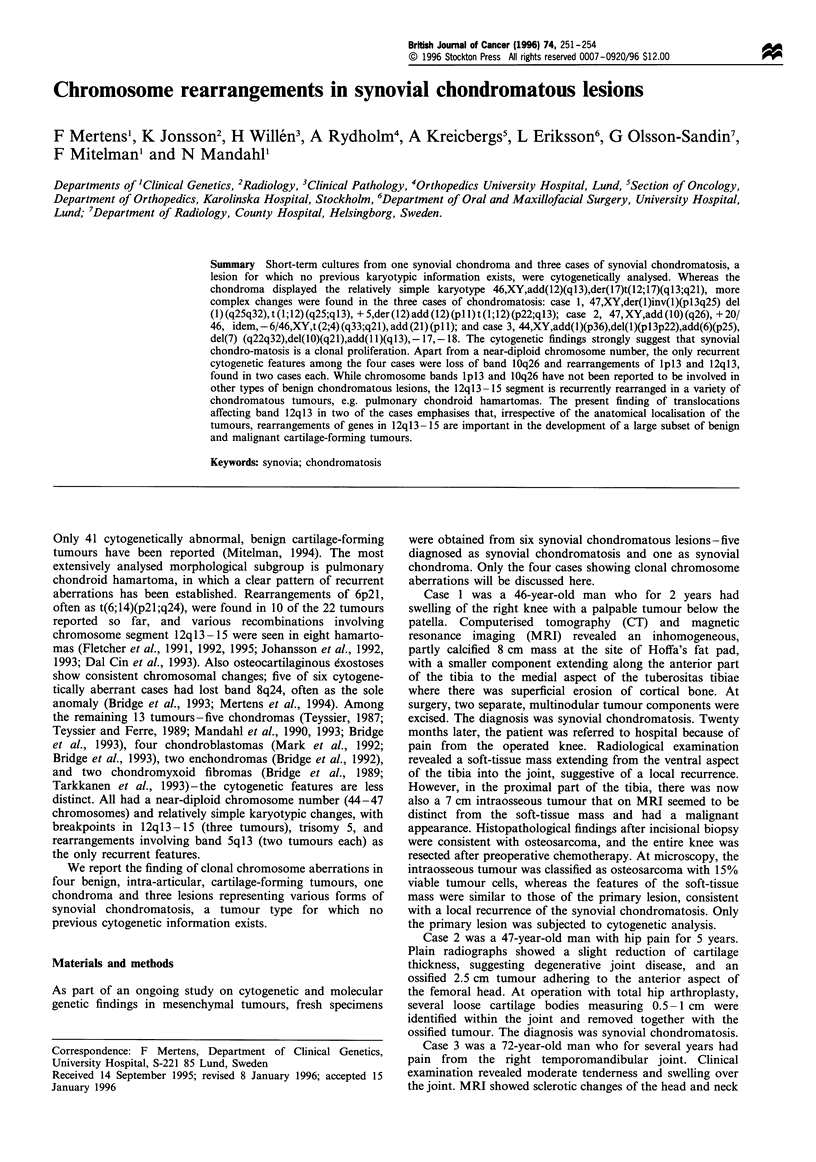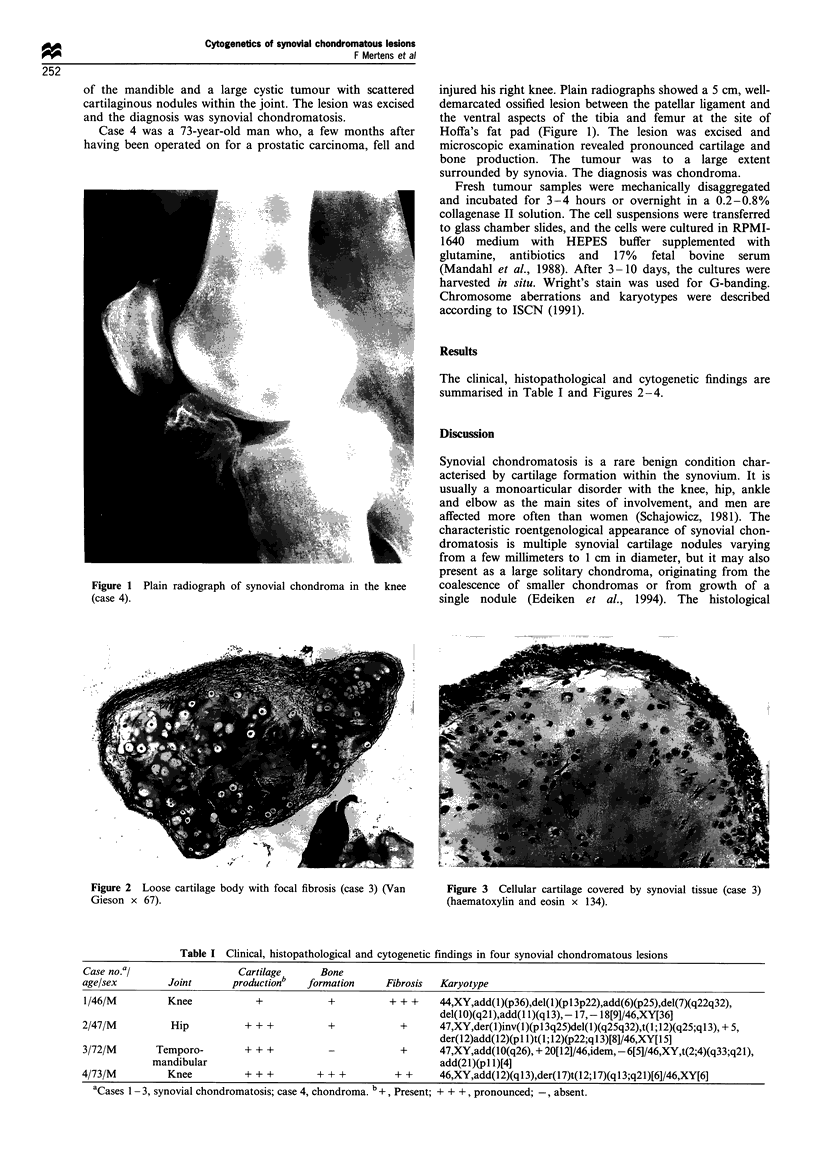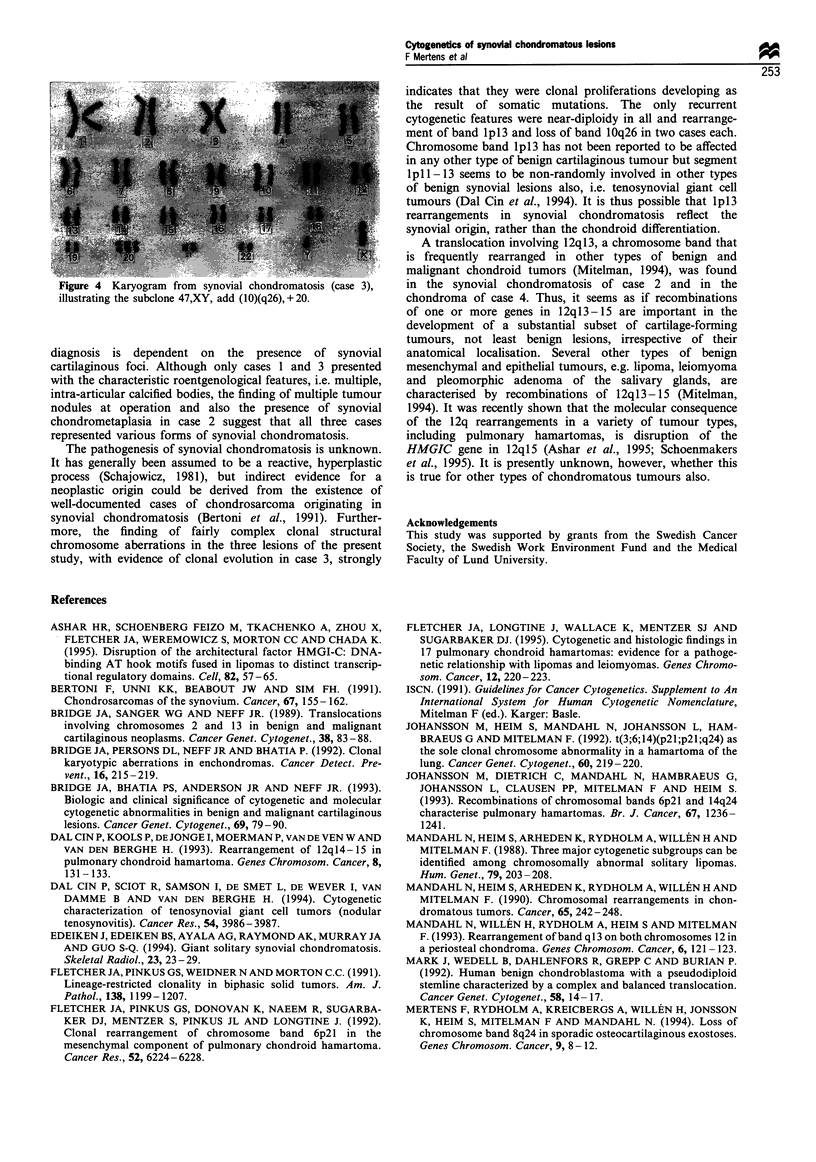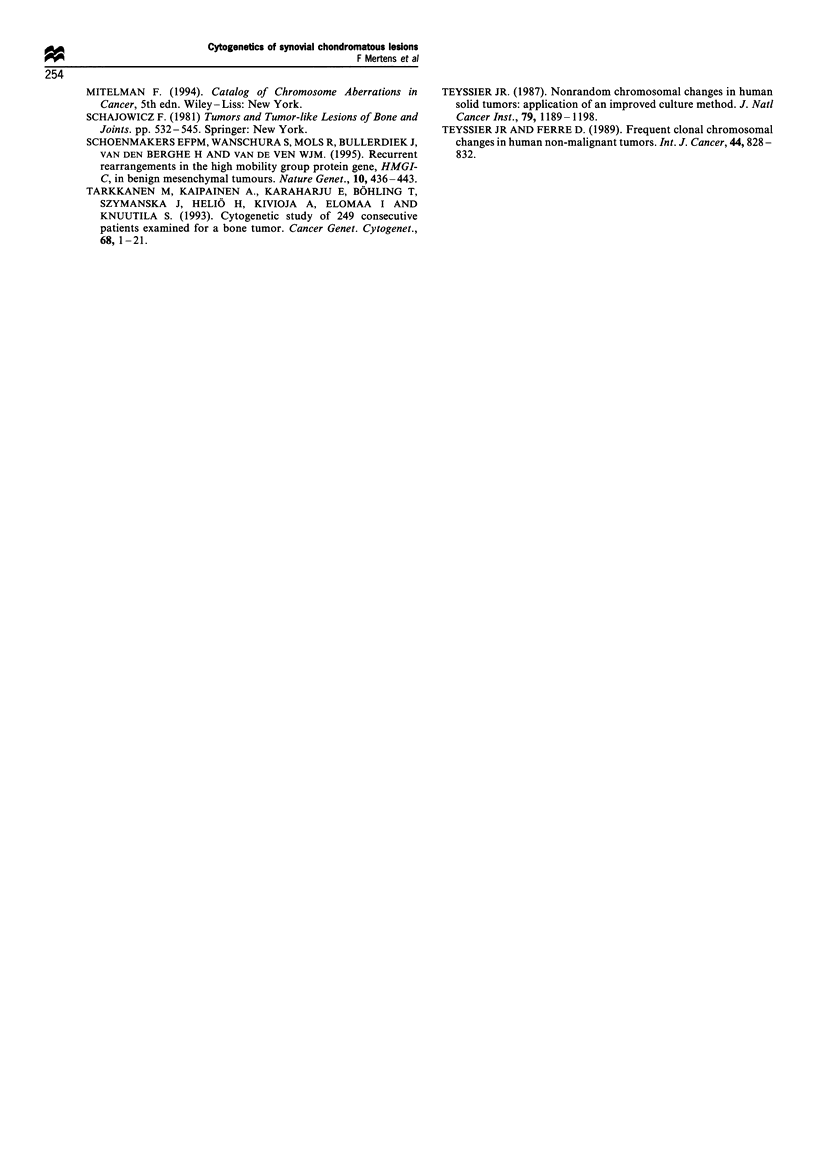# Chromosome rearrangements in synovial chondromatous lesions.

**DOI:** 10.1038/bjc.1996.346

**Published:** 1996-07

**Authors:** F. Mertens, K. Jonsson, H. Willén, A. Rydholm, A. Kreicbergs, L. Eriksson, G. Olsson-Sandin, F. Mitelman, N. Mandahl

**Affiliations:** Department of Clinical Genetics, University Hospital, Lund, Sweden.

## Abstract

**Images:**


British Journal of Cancer (1996) 74, 251-254
© 1996 Stockton Press All rights reserved 0007-0920/96 $12.00

### Chromosome rearrangements in synovial chondromatous lesions

F Mertens[1], K Jonsson[2], H Willén[3], A Rydholm[4], A Kreicbergs[5], L Eriksson[6], G Olsson-Sandin[7], F Mitelman[1] and N Mandahl[1]

*Departments of [1]Clinical Genetics, [2]Radiology, [3]Clinical Pathology, [4]Orthopedics University Hospital, Lund, [5]Section of Oncology, Department of Orthopedics, Karolinska Hospital, Stockholm, [6]Department of Oral and Maxillofacial Surgery, University Hospital, Lund; [7]Department of Radiology, County Hospital, Helsingborg, Sweden.*

**Summary** Short-term cultures from one synovial chondroma and three cases of synovial chondromatosis, a lesion for which no previous karyotypic information exists, were cytogenetically analysed. Whereas the chondroma displayed the relatively simple karyotype 46,XY,add(12)(q13),der(17)t(12;17)(q13;q21), more complex changes were found in the three cases of chondromatosis: case 1, 47,XY,der(1)inv(1)(p13q25) del (1)(q25q32),t(1;12)(q25;q13), +5,der(12)add(12)(p11)t(1;12)(p22;q13); case 2, 47,XY,add(10)(q26), +20/46, idem, −6/46,XY,t(2;4)(q33;q21),add(21)(p11); and case 3, 44,XY,add(1)(p36),del(1)(p13p22),add(6)(p25), del(7) (q22q32),del(10)(q21),add(11)(q13), −17, −18. The cytogenetic findings strongly suggest that synovial chondro-matosis is a clonal proliferation. Apart from a near-diploid chromosome number, the only recurrent cytogenetic features among the four cases were loss of band 10q26 and rearrangements of 1p13 and 12q13, found in two cases each. While chromosome bands 1p13 and 10q26 have not been reported to be involved in other types of benign chondromatous lesions, the 12q13−15 segment is recurrently rearranged in a variety of chondromatous tumours, e.g. pulmonary chondroid hamartomas. The present finding of translocations affecting band 12q13 in two of the cases emphasises that, irrespective of the anatomical localisation of the tumours, rearrangements of genes in 12q13−15 are important in the development of a large subset of benign and malignant cartilage-forming tumours.

**Keywords:** synovia; chondromatosis

Only 41 cytogenetically abnormal, benign cartilage-forming tumours have been reported (Mitelman, 1994). The most extensively analysed morphological subgroup is pulmonary chondroid hamartoma, in which a clear pattern of recurrent aberrations has been established. Rearrangements of 6p21, often as t(6;14)(p21;q24), were found in 10 of the 22 tumours reported so far, and various recombinations involving chromosome segment 12q13−15 were seen in eight hamartomas (Fletcher *et al.*, 1991, 1992, 1995; Johansson *et al.*, 1992, 1993; Dal Cin *et al.*, 1993). Also osteocartilaginous éxostoses show consistent chromosomal changes; five of six cytogenetically aberrant cases had lost band 8q24, often as the sole anomaly (Bridge *et al.*, 1993; Mertens *et al.*, 1994). Among the remaining 13 tumours−five chondromas (Teyssier, 1987; Teyssier and Ferre, 1989; Mandahl *et al.*, 1990, 1993; Bridge *et al.*, 1993), four chondroblastomas (Mark *et al.*, 1992; Bridge *et al.*, 1993), two enchondromas (Bridge *et al.*, 1992), and two chondromyxoid fibromas (Bridge *et al.*, 1989; Tarkkanen *et al.*, 1993)−the cytogenetic features are less distinct. All had a near-diploid chromosome number (44−47 chromosomes) and relatively simple karyotypic changes, with breakpoints in 12q13−15 (three tumours), trisomy 5, and rearrangements involving band 5q13 (two tumours each) as the only recurrent features.

We report the finding of clonal chromosome aberrations in four benign, intra-articular, cartilage-forming tumours, one chondroma and three lesions representing various forms of synovial chondromatosis, a tumour type for which no previous cytogenetic information exists.

### Materials and methods

As part of an ongoing study on cytogenetic and molecular genetic findings in mesenchymal tumours, fresh specimens were obtained from six synovial chondromatous lesions−five diagnosed as synovial chondromatosis and one as synovial chondroma. Only the four cases showing clonal chromosome aberrations will be discussed here.

Case 1 was a 46-year-old man who for 2 years had swelling of the right knee with a palpable tumour below the patella. Computerised tomography (CT) and magnetic resonance imaging (MRI) revealed an inhomogeneous, partly calcified 8 cm mass at the site of Hoffa's fat pad, with a smaller component extending along the anterior part of the tibia to the medial aspect of the tuberositas tibiae where there was superficial erosion of cortical bone. At surgery, two separate, multinodular tumour components were excised. The diagnosis was synovial chondromatosis. Twenty months later, the patient was referred to hospital because of pain from the operated knee. Radiological examination revealed a soft-tissue mass extending from the ventral aspect of the tibia into the joint, suggestive of a local recurrence. However, in the proximal part of the tibia, there was now also a 7 cm intraosseous tumour that on MRI seemed to be distinct from the soft-tissue mass and had a malignant appearance. Histopathological findings after incisional biopsy were consistent with osteosarcoma, and the entire knee was resected after preoperative chemotherapy. At microscopy, the intraosseous tumour was classified as osteosarcoma with 15% viable tumour cells, whereas the features of the soft-tissue mass were similar to those of the primary lesion, consistent with a local recurrence of the synovial chondromatosis. Only the primary lesion was subjected to cytogenetic analysis.

Case 2 was a 47-year-old man with hip pain for 5 years. Plain radiographs showed a slight reduction of cartilage thickness, suggesting degenerative joint disease, and an ossified 2.5 cm tumour adhering to the anterior aspect of the femoral head. At operation with total hip arthroplasty, several loose cartilage bodies measuring 0.5−1 cm were identified within the joint and removed together with the ossified tumour. The diagnosis was synovial chondromatosis.

Case 3 was a 72-year-old man who for several years had pain from the right temporomandibular joint. Clinical examination revealed moderate tenderness and swelling over the joint. MRI showed sclerotic changes of the head and neck

Correspondence: F Mertens, Department of Clinical Genetics, University Hospital, S-221 85 Lund, Sweden
Received 14 September 1995; revised 8 January 1996; accepted 15 January 1996

of the mandible and a large cystic tumour with scattered cartilaginous nodules within the joint. The lesion was excised and the diagnosis was synovial chondromatosis.

Case 4 was a 73-year-old man who, a few months after having been operated on for a prostatic carcinoma, fell and injured his right knee. Plain radiographs showed a 5 cm, well-demarcated ossified lesion between the patellar ligament and the ventral aspects of the tibia and femur at the site of Hoffa's fat pad (Figure 1). The lesion was excised and microscopic examination revealed pronounced cartilage and bone production. The tumour was to a large extent surrounded by synovia. The diagnosis was chondroma.

**Figure 1** Plain radiograph of synovial chondroma in the knee (case 4).

Fresh tumour samples were mechanically disaggregated and incubated for 3–4 hours or overnight in a 0.2–0.8% collagenase II solution. The cell suspensions were transferred to glass chamber slides, and the cells were cultured in RPMI-1640 medium with HEPES buffer supplemented with glutamine, antibiotics and 17% fetal bovine serum (Mandahl *et al.*, 1988). After 3–10 days, the cultures were harvested *in situ*. Wright's stain was used for G-banding. Chromosome aberrations and karyotypes were described according to ISCN (1991).

### Results

The clinical, histopathological and cytogenetic findings are summarised in Table I and Figures 2–4.

### Discussion

Synovial chondromatosis is a rare benign condition characterised by cartilage formation within the synovium. It is usually a monoarticular disorder with the knee, hip, ankle and elbow as the main sites of involvement, and men are affected more often than women (Schajowicz, 1981). The characteristic roentgenological appearance of synovial chondromatosis is multiple synovial cartilage nodules varying from a few millimeters to 1 cm in diameter, but it may also present as a large solitary chondroma, originating from the coalescence of smaller chondromas or from growth of a single nodule (Edeiken *et al.*, 1994). The histological

**Figure 2** Loose cartilage body with focal fibrosis (case 3) (Van Gieson × 67).

**Figure 3** Cellular cartilage covered by synovial tissue (case 3) (haematoxylin and eosin × 134).

**Table I** Clinical, histopathological and cytogenetic findings in four synovial chondromatous lesions

| *Case no.[a]/ age/sex* | *Joint* | *Cartilage production[b]* | *Bone formation* | *Fibrosis* | *Karyotype* |
|---|---|---|---|---|---|
| 1/46/M | Knee | + | + | + + + | 44,XY,add(1)(p36),del(1)(p13p22),add(6)(p25),del(7)(q22q32), del(10)(q21),add(11)(q13), – 17, – 18[9]/46,XY[36] |
| 2/47/M | Hip | + + + | + | + | 47,XY,der(1)inv(1)(p13q25)del(1)(q25q32),t(1;12)(q25;q13), + 5, der(12)add(12)(p11)t(1;12)(p22;q13)[8]/46,XY[15] |
| 3/72/M | Temporo-mandibular | + + + | – | + | 47,XY,add(10(q26), + 20[12]/46,idem, – 6[5]/46,XY,t(2;4)(q33;q21), add(21)(p11)[4] |
| 4/73/M | Knee | + + + | + + + | + + | 46,XY,add(12)(q13),der(17)t(12;17)(q13;q21)[6]/46,XY[6] |

[a]Cases 1–3, synovial chondromatosis; case 4, chondroma. [b]+, Present; + + +, pronounced; –, absent.

**Figure 4** Karyogram from synovial chondromatosis (case 3), illustrating the subclone 47,XY, add (10)(q26),+20.

diagnosis is dependent on the presence of synovial cartilaginous foci. Although only cases 1 and 3 presented with the characteristic roentgenological features, i.e. multiple, intra-articular calcified bodies, the finding of multiple tumour nodules at operation and also the presence of synovial chondrometaplasia in case 2 suggest that all three cases represented various forms of synovial chondromatosis.

The pathogenesis of synovial chondromatosis is unknown. It has generally been assumed to be a reactive, hyperplastic process (Schajowicz, 1981), but indirect evidence for a neoplastic origin could be derived from the existence of well-documented cases of chondrosarcoma originating in synovial chondromatosis (Bertoni *et al.*, 1991). Furthermore, the finding of fairly complex clonal structural chromosome aberrations in the three lesions of the present study, with evidence of clonal evolution in case 3, strongly indicates that they were clonal proliferations developing as the result of somatic mutations. The only recurrent cytogenetic features were near-diploidy in all and rearrangement of band 1p13 and loss of band 10q26 in two cases each. Chromosome band 1p13 has not been reported to be affected in any other type of benign cartilaginous tumour but segment 1p11–13 seems to be non-randomly involved in other types of benign synovial lesions also, i.e. tenosynovial giant cell tumours (Dal Cin *et al.*, 1994). It is thus possible that 1p13 rearrangements in synovial chondromatosis reflect the synovial origin, rather than the chondroid differentiation.

A translocation involving 12q13, a chromosome band that is frequently rearranged in other types of benign and malignant chondroid tumors (Mitelman, 1994), was found in the synovial chondromatosis of case 2 and in the chondroma of case 4. Thus, it seems as if recombinations of one or more genes in 12q13–15 are important in the development of a substantial subset of cartilage-forming tumours, not least benign lesions, irrespective of their anatomical localisation. Several other types of benign mesenchymal and epithelial tumours, e.g. lipoma, leiomyoma and pleomorphic adenoma of the salivary glands, are characterised by recombinations of 12q13–15 (Mitelman, 1994). It was recently shown that the molecular consequence of the 12q rearrangements in a variety of tumour types, including pulmonary hamartomas, is disruption of the *HMGIC* gene in 12q15 (Ashar *et al.*, 1995; Schoenmakers *et al.*, 1995). It is presently unknown, however, whether this is true for other types of chondromatous tumours also.

**Acknowledgements**
This study was supported by grants from the Swedish Cancer Society, the Swedish Work Environment Fund and the Medical Faculty of Lund University.

### References

ASHAR HR, SCHOENBERG FEIZO M, TKACHENKO A, ZHOU X, FLETCHER JA, WEREMOWICZ S, MORTON CC AND CHADA K. (1995). Disruption of the architectural factor HMGI-C: DNA-binding AT hook motifs fused in lipomas to distinct transcriptional regulatory domains. *Cell*, **82**, 57–65.

BERTONI F, UNNI KK, BEABOUT JW AND SIM FH. (1991). Chondrosarcomas of the synovium. *Cancer*, **67**, 155–162.

BRIDGE JA, SANGER WG AND NEFF JR. (1989). Translocations involving chromosomes 2 and 13 in benign and malignant cartilaginous neoplasms. *Cancer Genet. Cytogenet.*, **38**, 83–88.

BRIDGE JA, PERSONS DL, NEFF JR AND BHATIA P. (1992). Clonal karyotypic aberrations in enchondromas. *Cancer Detect. Prevent.*, **16**, 215–219.

BRIDGE JA, BHATIA PS, ANDERSON JR AND NEFF JR. (1993). Biologic and clinical significance of cytogenetic and molecular cytogenetic abnormalities in benign and malignant cartilaginous lesions. *Cancer Genet. Cytogenet.*, **69**, 79–90.

DAL CIN P, KOOLS P, DE JONGE I, MOERMAN P, VAN DE VEN W AND VAN DEN BERGHE H. (1993). Rearrangement of 12q14–15 in pulmonary chondroid hamartoma. *Genes Chromosom. Cancer*, **8**, 131–133.

DAL CIN P, SCIOT R, SAMSON I, DE SMET L, DE WEVER I, VAN DAMME B AND VAN DEN BERGHE H. (1994). Cytogenetic characterization of tenosynovial giant cell tumors (nodular tenosynovitis). *Cancer Res.*, **54**, 3986–3987.

EDEIKEN J, EDEIKEN BS, AYALA AG, RAYMOND AK, MURRAY JA AND GUO S-Q. (1994). Giant solitary synovial chondromatosis. *Skeletal Radiol.*, **23**, 23–29.

FLETCHER JA, PINKUS GS, WEIDNER N AND MORTON C.C. (1991). Lineage-restricted clonality in biphasic solid tumors. *Am. J. Pathol.*, **138**, 1199–1207.

FLETCHER JA, PINKUS GS, DONOVAN K, NAEEM R, SUGARBAKER DJ, MENTZER S, PINKUS JL AND LONGTINE J. (1992). Clonal rearrangement of chromosome band 6p21 in the mesenchymal component of pulmonary chondroid hamartoma. *Cancer Res.*, **52**, 6224–6228.

FLETCHER JA, LONGTINE J, WALLACE K, MENTZER SJ AND SUGARBAKER DJ. (1995). Cytogenetic and histologic findings in 17 pulmonary chondroid hamartomas: evidence for a pathogenetic relationship with lipomas and leiomyomas. *Genes Chromosom. Cancer*, **12**, 220–223.

ISCN. (1991). *Guidelines for Cancer Cytogenetics. Supplement to An International System for Human Cytogenetic Nomenclature*, Mitelman F (ed.). Karger: Basle.

JOHANSSON M, HEIM S, MANDAHL N, JOHANSSON L, HAMBRAEUS G AND MITELMAN F. (1992). t(3;6;14)(p21;p21;q24) as the sole clonal chromosome abnormality in a hamartoma of the lung. *Cancer Genet. Cytogenet.*, **60**, 219–220.

JOHANSSON M, DIETRICH C, MANDAHL N, HAMBRAEUS G, JOHANSSON L, CLAUSEN PP, MITELMAN F AND HEIM S. (1993). Recombinations of chromosomal bands 6p21 and 14q24 characterise pulmonary hamartomas. *Br. J. Cancer*, **67**, 1236–1241.

MANDAHL N, HEIM S, ARHEDEN K, RYDHOLM A, WILLÉN H AND MITELMAN F. (1988). Three major cytogenetic subgroups can be identified among chromosomally abnormal solitary lipomas. *Hum. Genet.*, **79**, 203–208.

MANDAHL N, HEIM S, ARHEDEN K, RYDHOLM A, WILLÉN H AND MITELMAN F. (1990). Chromosomal rearrangements in chondromatous tumors. *Cancer*, **65**, 242–248.

MANDAHL N, WILLÉN H, RYDHOLM A, HEIM S AND MITELMAN F. (1993). Rearrangement of band q13 on both chromosomes 12 in a periosteal chondroma. *Genes Chromosom. Cancer*, **6**, 121–123.

MARK J, WEDELL B, DAHLENFORS R, GREPP C AND BURIAN P. (1992). Human benign chondroblastoma with a pseudodiploid stemline characterized by a complex and balanced translocation. *Cancer Genet. Cytogenet.*, **58**, 14–17.

MERTENS F, RYDHOLM A, KREICBERGS A, WILLÉN H, JONSSON K, HEIM S, MITELMAN F AND MANDAHL N. (1994). Loss of chromosome band 8q24 in sporadic osteocartilaginous exostoses. *Genes Chromosom. Cancer*, **9**, 8–12.

MITELMAN F. (1994). *Catalog of Chromosome Aberrations in Cancer*, 5th edn. Wiley–Liss: New York.

SCHAJOWICZ F. (1981) *Tumors and Tumor-like Lesions of Bone and Joints*. pp. 532–545. Springer: New York.

SCHOENMAKERS EFPM, WANSCHURA S, MOLS R, BULLERDIEK J, VAN DEN BERGHE H AND VAN DE VEN WJM. (1995). Recurrent rearrangements in the high mobility group protein gene, *HMGI-C*, in benign mesenchymal tumours. *Nature Genet.*, **10,** 436–443.

TARKKANEN M, KAIPAINEN A., KARAHARJU E, BÖHLING T, SZYMANSKA J, HELIÖ H, KIVIOJA A, ELOMAA I AND KNUUTILA S. (1993). Cytogenetic study of 249 consecutive patients examined for a bone tumor. *Cancer Genet. Cytogenet.*, **68,** 1–21.

TEYSSIER JR. (1987). Nonrandom chromosomal changes in human solid tumors: application of an improved culture method. *J. Natl Cancer Inst.*, **79,** 1189–1198.

TEYSSIER JR AND FERRE D. (1989). Frequent clonal chromosomal changes in human non-malignant tumors. *Int. J. Cancer*, **44,** 828–832.